# Knowledge of non-communicable diseases and practices related to healthy lifestyles among adolescents, in state schools of a selected educational division in Sri Lanka

**DOI:** 10.1186/s12889-017-4622-z

**Published:** 2017-07-26

**Authors:** A. U. Gamage, P. L. Jayawardana

**Affiliations:** 1 Consultant Community Physician, Ministry of Health, 385, Ven. Baddegama Wimalawansa Thero Mawatha, Colombo, 10 Sri Lanka; 2 Professor in Public Health, Department of Public Health, Faculty of Medicine, University of Kelaniya, Ragama, Sri Lanka

**Keywords:** Non-communicable disease, Knowledge, Lifestyle practices, Prevention, Adolescents

## Abstract

**Background:**

Behaviors established during the adolescence have life-long consequences to the onset of non-communicable diseases (NCDs) in later life. Therefore, it is essential to understand adolescents’ knowledge and practices with the intention of developing preventive programs focusing on this age group. The objective of the study was to assess knowledge about selected NCDs, and lifestyle choices among school students aged 17–19 years in state schools of the Maharagama Educational Division, Sri Lanka.

**Methods:**

A descriptive, cross-sectional study was conducted among students aged 17–19 years attending state schools in Maharagama Education Division. A total of 634 students were selected from 9 schools conducting Advance Level classes. Stratified sampling was done based on stream of study and the number needed from each stratum was decided according to probability proportionate to size which was followed by cluster sampling within the strata to select the classes included.

Data were collected using a self-administered-questionnaire on socio-demographic characteristics and economic status; lifestyle-related practices; knowledge on Non-Communicable -Diseases. Logistic regression was used to assess the associations.

**Results:**

Proportion students with good overall knowledge was 43%(*n* = 272). Forty-three percent (*n* = 275) consumed a healthy diet, and 20%(*n* = 129) engaged in adequate physical activity 3%(*n* = 18) of students were current smokers and 12%(*n* = 73) current alcohol users 12%(*n* = 73).

Overall “good” knowledge about NCDs was associated with being a science stream student(OR = 3.3; 95%CI:2.1–5.2). Healthy diet was associated with female sex (OR = 2.1; 95%CI: 1.5–3.0), and adequate physical activity with male sex (OR = 2.1; 95% CI:1.4–3.2), non-science-stream (OR = 2.1; 95%CI:1.2–3.7) and upper socio economic status (OR = 2.0; 95%CI:1.3–3.0). Non-smoking was associated with overall good knowledge (OR = 4.1; 95%CI:1.2–13.7) and female sex (OR = 0;95%CI:1.5-infinity). Abstinence from alcohol was associated with being a female (OR = 6.9; 95%CI:3.4–13.9), and with mother and fathers’ education level of > General-Certificate of Examinations Ordinary Level (GCE O/L) (OR = 2.9; 95%CI:1.1–8.4 and OR = 3.5; 95%CI:1.1–11.2 respectively).

**Conclusion:**

Knowledge about NCDs and healthy lifestyle-practices were poor among school students aged 17–19 years. Lack of knowledge about healthy and unhealthy behaviors highlights the importance of carrying out regular surveillance for NCD risk factors, and initiating programs for the prevention of NCDs amongst adolescents.

**Electronic supplementary material:**

The online version of this article (doi:10.1186/s12889-017-4622-z) contains supplementary material, which is available to authorized users.

## Background

Non-communicable diseases (NCDs) are diseases that are not transmissible from one person to another. [1] Commonly existing NCDs include hypertension, diabetes mellitus, cardiovascular diseases (coronary heart disease and stroke), cancers, injuries and chronic respiratory disease. Among the main contributing factors are older age and unhealthy lifestyle-related behaviours, hence the term “lifestyle-related diseases.” A general improvement of socio-economic factors within Sri Lanka and the resulting increase in life expectancy, has contributed to an increase in the prevalence of NCDs. [2, 3].

Adolescents are classified as individuals between the ages of 10 to 19 years. [4] In Sri Lanka, 19.7% of the population (3.7 million people) are adolescents; a majority of whom (72.9%) are school goers. [5] It is a well-known fact that most lifestyle-related risk factors for NCDs are laid down during this period. [5, 6] This calls for action to prevent the establishment of risk factors from the early years of life; by arming adolescents with adequate knowledge about chronic diseases and healthy preventive practices. Evidence on the current knowledge and lifestyle-related practices of adolescents would immensely help both the health and education sectors with planning and implementation of much needed programs for school children. Educating students at the school level is considered to have a significant impact on the prevention of NCDs. [5, 7].

The present study aims to determine the level of knowledge about selected NCDs and the level of engagement in healthy behaviours, among school students aged 17–19 years in state schools of the Maharagama Educational Division in the district of Colombo, Sri Lanka. Specifically, it will assess knowledge levels on coronary heart disease, diabetes mellitus, hypertension, cerebrovascular disease, and cancers, as well as knowledge about the relevant primary preventive measures for these diseases including the lifestyle measures, which have been adopted to prevent the above NCDs.

## Methods

### Participants

A descriptive cross-sectional study was conducted among students aged 17 to 19 years, in General-Certificate of Examinations Advanced Level class, attending state schools in the Maharagama education division, of the Colombo district, Sri Lanka. The study was conducted in the Western Province (one of nine provinces in the country), which is further divided into three districts (Colombo, Gampaha, and Kalutara). The Education Department has divided each district into education zones, with Maharagama education division one of the four such zones within the Colombo district. Of the 1359 national and provincial schools in the Western Province, 406 are located in the Colombo district. Among the 47,179 students enrolled for the General-Certificate of Examinations Advanced Level (GCE A/L classes) (year 12 and 13) country-wide in 2010, 13,301 were in Arts, 18,955 in Commerce, and 14,923 in Science streams. [8].

In total, the number of schools conducting GCE A/L classes in the Maharagama division was nine, with 1044 year-12 and 13 students distributed within the three streams of study (Arts, Commerce, and Science).

Based on the formula proposed by Lwanga, Lemeshow, [9] the initial sample size calculated was 384, having considered a proportion with adequate knowledge and practices for each as 50%. This sample size was subsequently inflated with a design effect set at 1.5 and addition of a non-response rate of 5%. The resulting sample size, of 605, was then further inflated to obtain integer division with the cluster size set at 25 (the minimum number of students available in the classes selected for the study). Thus, the final sample size computed was 625 with a total of 25 (625/25) clusters selected from the nine schools.

The required number of classes from each stratum was calculated by applying probability proportion to the size of the student population in each stratum, which was 22%, 32% and 45% in the Science, Arts, and Commerce streams, respectively. Thus the number of classes required were selected randomly resulting in eight Science: eleven Arts, and 16 Commerce classes, respectively. Despite the calculated sample size, all students in a selected class (the number of students in a given class ranged from 25 to 35) were included due to ethical requirements, with the final number of students recruited to the study and included in the analysis as 634.

### Study instrument

A self-administered-questionnaire was used to gather data from recruited participants. The questionnaire consisted of four components: socio-demographic characteristics and socio-economic status; lifestyle-related practices; knowledge on NCDs, and sources of information. The designing of the latter was based on the Pan American Health Organization (PAHO) NCD surveillance toolkit, [10] and the STEP-wise-approach-to-Surveillance (STEPS) of NCD risk factor questionnaire. [11] The socio-economic status component was based on a questionnaire to assess socio-economic index, resulting in a composite score. [12] The Questionnaire is provided in Additional file 1.

When preparing the questionnaire, the use of technical terms was kept to a minimum to retain clarity, particularly for the benefit of Commerce and Arts students who took part in the study. Questionnaires were provided anonymously, to maximize accuracy and reliability concerning the responses obtained. The questionnaire was developed in Sinhala language and pre-tested among 15 GCE A/L students representing all three-study streams from a different educational division, to avoid the influence of testing effect.

Students’ anthropometric measurements were collected by research staff and recorded in a data extraction sheet. Weight (using a digital weighing scale) and height (using a microtoise steel tape) were obtained using standard techniques, rounding to the nearest 100 g for weight, and 0.5 cm for height.

### Data analysis

#### Analysis of practices

Assessment of the level of physical activity was based upon those who gave a positive response to being engaged in some form of moderate to vigorous physical activity. They were further classified as those with adequate/inadequate physical activity based on the following criteria: time duration of exercise ≥60 min on a daily basis. [13] The physical activity was considered adequate only if both criteria were fulfilled.

A student was considered to have healthy dietary habits if the following criteria were met: Consuming 1) home prepared breakfast for ≥5 days/week; 2) non-fast food for breakfast for ≥5 days/ week; 3) fruits ≥5 days/week; 4) ≥5 servings/day of vegetables and green leaves 5) consuming fruit juice ≥5 days/week; 6) carbonated drinks infrequently (<2 days/week), and 7) non-consumption of additional salt (in comparison to their other family members). Each fulfilled criterion was given one point, with a maximum possible score of seven. Those who obtained a score ≥ 5 were considered as having healthy dietary habits. The dietary questionnaire used for the current study was not validated previously.

A smoker was defined as one who smokes currently. Those who had previously (but not currently), or never smoked were considered as non-smokers. A student who was currently consuming alcohol was considered as ‘consuming alcohol, ‘and those who had previously (but not currently), or never consumed were considered as “not consuming alcohol.”

#### Analysis of knowledge

This section consisted of 39 questions related to student’s knowledge about NCDs and their risk factors. Correct answers were given a score of 1 and incorrect answers given 0. The total possible score ranged from 0 to 216 and was expressed as a percentage the total possible score. Components and assignment of the score for knowledge is provided in Additional file 2. A cut-off level ≥ 60%, the median value of the individual percentage scores, was selected as an indicator of “good” knowledge.

#### Socio-economic status

Socio-economic status was based upon a composite score, developed and validated for the Sri Lankan setting, comprising separate scores of eight individual variables (father’s education status, mother’s education status, father’s occupation status, mother’s occupation status, material used for the floor, availability of latrine facility, source of drinking water, possessions of items by the family members). [12] The sample was divided into quintiles based on the individual composite score computed, and assigned accordingly to five socio-economic classes. For the analysis of associations, the top two (1st and 2nd quintiles of higher social classes) and lower three quintiles (3rd-5th quintiles of lower social classes) were amalgamated.

#### Body mass index

Body mass index (BMI) was calculated for each student using his or her weight and height measurement. The cutoff values for BMI were based on World Health Organization (WHO) reference values for adolescents. [14, 15].

We used SPSS [16] data analysis and statistical software to report data as the means ± SD and medians for continuous variables and proportions, then percentages for categorical values. Bivariate analyses were conducted using chi-square test. The level considered significant was a probability (p) value of less than 0.05. The factors, which had a *p*-value of ≤0.25, and the biologically plausible ones, despite having a *p* value of >0.25 were included in a multiple logistic regression analysis. List wise deletion method was used for missing data. Associations were expressed as odds ratios (OR) and their respective 95% confidence intervals (95% CI).

## Results

The computed sample size was 625, and the number of students in the selected classes was 696. Of these, 62 were absent, giving a non-response rate of 8.9%. The total number of students included in the study was 634; all of whom were included in the analyses. The distribution of students by study stream was 41.2% (*n* = 243) from Arts, 38.2% (*n* = 261) from Commerce and 20.5% (*n* = 130) from Science.

### Demographic and socioeconomic characteristics

The mean age of the sample was 18.4 (SD = 0.38) years, 45.3% (*n* = 287) of which were male students. 626 (98.7%) were Sinhalese. Tamil and Moors were each 0.5% (*n* = 3) and Burghers 0.3% (*n* = 2) [Table 1]. The highest proportion of fathers (22.9%: *n* = 145) and mothers (29%; *n* = 184) had passed the General Certificate of Education; Ordinary Level (GCE O/L) examination. Concerning the socio-economic status (SES), the highest proportion (19.6%: *n* = 124) belonged to the richest quintile (5th quintile).

**Table 1:** Frequency distribution of demographic and socioeconomic. Characteristics of the advanced level students

Variable^a^	*Females*	*Males*	*Total (Row)*
Total	347(54.7%)	287(45.3%)	634 (100%)
	n (%)	n (%)	n (%)
Ethnicity			
Sinhalese	341 (98.2)	285 (99.3)	626 (98.7)
Tamil	3 (0.9)	0 (0)	3 (0.5)
Moors	3 (0.9)	0 (0)	3 (0.5)
Burger	0 (0)	2 (0.7)	2 (0.3)
Father’s level of education			
No schooling	2 (0.6)	0 (0)	2 (0.3)
Grade 1–5	15 (4.3)	6 (2.1)	21 (3.3)
Grade 6–10	63 (18.2)	41 (14.3)	104 (16.4)
Passed GCE O/L^a^	72 (20.7)	73 (25.4)	145 (22.9)
Up to GCE A/L^b^	56 (16.2)	46 (16.0)	102 (16.1)
Passed GCE A/L	74 (21.3)	63 (22.0)	137 (21.6)
University/Professional degree	16 (4.6)	28 (9.8)	44 (6.9)
Not known/ Missing	49 (14.1)	30 (10.4)	79 (12.5)
Mother’s level of education			
No schooling	2 (0.6)	1 (0.3)	3 (0.5)
Grade 1–5	14 (4.0)	9 (3.1)	23 (3.6)
Grade 6–10	54 (15.6)	39 (13.6)	93 (14.7)
Passed GCE O/L	107 (30.8)	77 (26.9)	184 (29.0)
Up to GCE A/L	63 (18.2)	58 (20.2)	121 (19.1)
Passed GCE A/L	77 (22.2)	67 (23.3)	144 (22.7)
University/Professional degree	4 (1.2)	13 (4.5)	17 (2.7)
Not known/ Missing	26 (7.4)	23 (8.1)	49 (7.7)
Socioeconomic status			
First Quintile-poorest	73 (21.0)	29 (10.1)	102 (16.1)
Second Quintile	43 (12.4)	43 (15.0)	86 (13.6)
Third Quintile	51 (14.7)	52 (18.2)	103 (16.2)
Fourth Quintile	52 (15.0)	51 (17.8)	103 (16.2)
Fifth quintile-richest	59 (17.0)	65 (22.5)	124 (19.6)
Not known/ Missing	69 (19.9)	47 (16.4)	116 (18.3)
Mean age (SD) years	17.9 (0.56)	18.9 (0.5)	18.4 (0.38)

^a^General Certificate of Education; Ordinary Level Examination

^b^General Certificate of Education; Advanced Level Examination

### Practices

The proportion of students involved in adequate physical activity among those engaged in physical activity was 31.8% (129/406) and among all students was 20.3% (129/634). The majority of all students (73.3%: *n* = 465) consumed breakfast prepared at home for ≥5 days/week, and 60.9% (*n* = 386) consumed healthy food (rice or rice-flour based food) for breakfast most of the week (≥5 days/week). However, 8.1% (*n* = 52) reported consuming bread or wheat flour based products for ≥5 days/week (Table 2).

**Table 2:** Frequency distribution of behavioral risk factors of the Advanced level students

No.	Variable	Frequency (%)		
		Males	Females	Total
1.	Physical activity (*N* = 634)			
	Yes	208(72.5%)	198(57.1%)	406 (64.0%)
	No	79(27.5%)	149 (42.9%)	228 (36.0%)
2.	Physical activity: adequacy among those who engaged in it (*N* = 406)			
	Adequate	79(38.0%)	50 (25.3%)	129 (31.8%)
	Inadequate	129 (62.0%)	148 (74.7%)	277 (68.2%)
3.	Type of physical activity (*N* = 421)^a^			
	Sports	148(71.1%)	78(39.4%)	226 (55.7%)
	Aerobics	24 (11.5%)	12 (6.1%)	36 (8.9%)
	Yoga	30 (14.4%)	32 (16.2%)	62 (15.3%)
	Dancing	11 (5.2%)	80 (40.4%)	91 (22.4%)
	Gardening, housekeeping	2 (0.9%)	4 (2.0%)	6 (1.5%)
4.	Dietary habits (*N* = 634)			
	Unhealthy dietary habits	193(67.2%)	166(47.8%)	359 (56.6%)
	Healthy dietary habits	94(32.8%)	181(52.2%)	275 (43.4%)
5.	Smoking (*N* = 652)^b^			
	Ever users	43(15.0%)	2 (0.6%)	45 (7.1%)
	Current users	18 (6.3%)	0 (0.0%)	18 (2.8%)
	Never users	244(85.0%)	345(99.4%)	589 (92.9%)
6.	Alcohol consumption (*N* = 707)^c^			
	Ever users	89(25.6%)	24(6.7%)	113 (16.0%)
	Current users	61(17.5%)	12 (3.3%)	73 (10.3%)
	Never users	198(56.9%)	323(90.0)	521 (73.7%)
7.	Type of alcohol consumed by current users (*n* = 127)^d^			
	Beer	44(72.1%)	12(100%)	56 (76.7%)
	Arrack	21(34.4%)	0(0.0%)	21 (28.8%)
	Brandy	10 (16.4%)	1(8.3%)	11 (15.1%)
	Jin	14(22.9%)	0(0.0%)	14 (19.2%)
	Wine	10(16.4%)	5(41.7%)	15 (20.5%)
	Whisky	10(16.4%)	0(0.0%)	10 (13.7%)
8.	Obesity/Over weight (*N* = 634)			
	Obese/ Over weight	43(15.0%)	28(8.1%)	71 (11.1%)
	Normal/Under-weight	244(85.0%)	319(91.9%)	563 (88.9%)

^a^Type of physical activity – total exceeds 406 (refer row no. 1) as 15 were engaged in more than one type of exercise

^b^Smoking – Total exceeds 634 as 18 current users were included under “ever users”

^c^Alcohol consumption – Total exceeds 634 as 73 current users were included under “ever users”

^d^Type of alcohol consumed by current users – total exceeds 73 (refer row no. 7) as 54 consumed more than one type of alcohol

A majority of all students (92.7%; *n* = 588) did not consume more salt than their family members. 65%(*n* = 413) consumed more fresh-fruit drinks than artificial ones (31.5%; *n* = 200). Only 24.1% (*n* = 153) reported consuming fruits for ≥5 days, and 31.7% (*n* = 201) reported ≥5 servings of vegetables/day. Based on the cut-off value of a score of ≥5, 52.2% (*n* = 181) of females and 32.8% (*n* = 94) of males had healthy dietary habits.

The proportion of current smokers was 2.8% (*n* = 18), and all were males. Among them, three (0.5%) students were daily smokers. The mean number of cigarettes smoked per day was 2.1 (SD = 1.3). The mean age at commencement of smoking was 16 (SD = 0.8) years.

The proportion of students currently consuming alcohol was 11.5% (*n* = 73), and among them, 83.5% (61/73) were males. 89 (25.6%) males and 24 (6.7%) females reported consuming alcohol previously. The mean age at commencement of consuming alcohol was 16.1 (SD = 1.3) years.

According to the computed BMI values, 49.8% (*n* = 316) of the students were underweight. More females were underweight (*n* = 183; 52.7%) than males (*n* = 133; 46.3%). The proportion of overweight was 6.6% (*n* = 42) and obesity 4.6% (*n* = 29). Obesity and overweight was significantly more prevalent in males (*n* = 43; 15%) than females (*n* = 28; 8.1%);(*p* < 0.005).

### Level of knowledge

Forty-three percent (42.9%; *n* = 272) of students obtained a score of ≥60%, (mean;119.7(SD = 31.0), median;124, IQR;99.7to141) for overall knowledge.

When individual subject areas were considered, a higher proportion of students had “good” knowledge on coronary artery disease (56.3%; *n* = 356) (mean;13.4 (SD = 4.7), median;14, IQR; 11 to 17), diet (74.9%; *n* = 475) (mean;25.9 (SD = 6.6), median;27, IQR;22 to 31) and physical activity (56.6%; *n* = 359) (mean;6.8 (SD = 2.9), median;7,IQR; 5 to 9) [Table 3].

**Table 3:** Distribution of knowledge scores on selected Non Communicable Diseases

Score on knowledge	Frequency (%) *N* = 634	Median	Mode	Mean (SD)
Non-Communicable Diseases: Total Score = 11				
<60%	372 (58.7%)	5	9	5.5 (3.3)
≥60%	262 (41.3%)			
Diabetes mellitus: Total Score = 31				
<60%	420 (66.2%)	16.5	18	16.0 (5.2)
≥60%	214 (33.8%)			
Hypertension: Total Score = 24				
<60%	516 (81.4%)	10	9	10.4 (4.5)
≥60%	118 (18.6%)			
Coronary artery disease: Total Score = 23				
<60%	277 (43.7%)	14	15	13.4 (4.7)
≥60%	357 (56.3%)			
Stroke: Total Score = 23				
<60%	418 (65.9%)	11	14	11.3 (6.6)
≥60%	216 (34.1%)			
Cancers: Total Score = 34				
<60%	391 (61.7%)	19	21	18.2 (6.0)
≥60%	243 (38.3%)			
Diet: Total Score = 37				
<60%	159 (25.1%)	27	31	25.9 (6.6)
≥60%	475 (74.9%)			
Physical activity: Total Score = 11				
<60%	275 (43.4%)	7	10	6.8 (2.9)
≥60%	359 (56.6%)			
Smoking: Total Score = 10				
<60%	324 (51.1%)	5	5	5.4 (1.8)
≥60%	310 (48.9%)			
Alcohol Consumption: Total Score = 12				
<60%	371 (58.5%)	7	7	6.9 (3.2)
≥60%	263 (41.5%)			
Overall: Total Score = 216				
<60%	362 (57.1%)	124	139	119.7 (31.0)
≥60%	272 (42.9%)			

Of the student who scored ≥60%, majority were females (*n* = 148; 54.4%), 265 (97.4%) were Sinhalese students. A majority (*n* = 85, 65.4%) of the science students scored ≥60%. Considering parent’s education level and “good overall knowledge,” 205 (80.4%) student’s whose father’s had an education of ≥ GCE O/L obtained a score of ≥60%. Similarly, Majority of the students (*n* = 209; 80.0%) of who’s mothers had an education of ≥ GCE O/L obtained a score of ≥60%. Hundred and thirty-eight students (56.6%) from lower socio-economic strata scored ≥60% compared to the higher SES students. Overall better knowledge was significantly associated with race, father’s education level (*p* < 0.1) and the stream of study p (0.001).

### Associated factors

Based on the multivariate analysis, a good overall knowledge on NCDs was associated with being a science stream student (OR = 3.3; 95%CI: 2.1–5.2) [Table 4].

**Table 4:** Factors significantly associated with knowledge

Model	Unadjusted (95% CI)		Adjusted ^a^ (95% CI)	
*Knowledge - Overall Score ≥ 60%*				
Science stream	3.2	2.1–4.8	3.3	2.1–5.2

^a^ *adjusted for Sex, stream of study, Fathers education level, Mothers education level, Socio-economic status*

A healthy diet (Model1) was significantly associated with female sex (OR = 2.1; 95% CI:1.5–3.0) only [Table 5]. Adequate physical activity (Model 2) was associated with male sex (OR = 2.1; 95% CI:1.4–3.2), non-science study streams (OR = 2.1; 95% CI: 1.2–3.7) and upper social SES (OR = 2.0; 95% CI:1.3–3.0) [Table 5]. Abstinence from smoking (Model 3) was associated with female sex (OR = 0; 95% CI: 1.5-infinity) and having good overall knowledge on NCDs (OR = 4.1; 95% CI:1.2–13.7). Abstinence from alcohol (Model 4) was associated with female sex (OR = 6.9; 95% CI:3.4–13.9), and mother and fathers’ educational level greater than GCE O/L (OR = 2.9; 95% CI:1.1–8.4 and OR = 3.5; 95% CI:1.1–11.2 respectively) [Table 5].

**Table 5:** Factors significantly associated with Practices

Model	Unadjusted (95% CI)		Adjusted ^b^ (95% CI)	
*Model 1-Healthy Diet*				
Female	3.2	2.1–4.8	3.3	2.1–5.2
*Model 2-Adequate Physical Activity*				
Male	2.3	1.5–3.6	2.1	1.4–3.2
Non-Science	1.6	0.9–2.8	2.1	1.2–3.7
Upper SES	2.0	1.3–3.0	2.0	1.3–3.0
*Model 3- Abstinence from Smoking*				
Females	0	5.6-Infinity	0	1.5-Infinity
Good overall knowledge	2.1	0.8–5.6	4.1	1.2–13.7
*Model 4- Abstinence from Consumption of Alcohol*				
Females	3.9	3.9–15.7	6.9	3.4–13.9
Mothers’ education >GCE (O/L)^a^	1.1	0.6–2.0	2.9	1.1–8.4
Father’s education >GCE (O/L)	1.9	0.9–3.9	3.5	1.1–11.2

^a^General Certificate of Education Ordinary Level

^b^ *adjusted for Sex, Stream of study, Fathers Education level, Mothers Education level, Socio economic status, Knowledge on NCDs*

## Discussion

Results of the present study indicate that the overall knowledge (42.9%; 95% CI: 39.0–46.9%) on NCDs and lifestyle-practices among the study group was poor. Despite the above, a higher proportion (56.2%) of students had good knowledge on coronary artery disease, healthy diet (74.9%) and physical activity (56.6%) as protective factors. In comparison to knowledge, the proportion practicing overall adequate physical activity was only 20.3% (*n* = 129). However previous studies have reported that awareness of PA recommendations and the actual level of PA are significantly associated. [17, 18] There is consensus in the literature that physical activity declines markedly during adolescence. [19] This might explain the current finding that irrespective of the awareness the proportion involved in adequate PA is low.

The overall knowledge of diabetes hypertension and other NCDs were poor among students. The poor knowledge might be dangerous because these are the individuals who are about to enter the society and to fight the epidemic of NCDs, and should be knowledgeable enough of the diseases and their prevention. The good knowledge of coronary artery disease might be from family members whose suffering from the disease and a good knowledge on diet, and physical activity would be due to the high level of publicity given to them to promote these.

Overall good knowledge about NCDs was higher among science stream students. Although kept to a minimum, it is possible the use of technical terms may have been factor contributing to the poor knowledge demonstrated among non-science stream students. This highlights the importance of addressing such deficiencies during the preparation of health education materials, in order to ensure messages reach all equally. Given the poor knowledge observed among the students of the highest school grade, the need for quick action becomes evident.

Less than one-third of students reported consuming adequate quantities of vegetables/green leaves (32%) and fruits (24%). This finding is comparable with the results reported by Vithana, [20] in a study conducted among teachers in Kegalle Medical Officer of Health area, Sri Lanka. The best strategy to encourage adoption of a healthy diet appears to be through a school-based policy, combined with awareness programs and participatory approach of both students and the community. [7, 21] However, a majority of students (73.3%) consumed a home-prepared breakfast, the finding of which is also consistent with the study reported by Kumarapeli. [22] Individual practices related to diet may be dependent on family food choices as well as availability, accessibility, and affordability which are influenced by the fluctuations in cost due to weather conditions experienced throughout the year.

Various cutoffs for adequate physical activity levels among adolescents have been used in previous studies. [23] The current application of adult physical activity guidelines for adolescents makes it difficult to accurately categorise physical activity levels among them. [24] The current study considered adequate physical activity levels as fulfilling both a time duration of ≥60 min, on a daily basis [13, 25], Based on this cutoff, physical activity was inadequate among a majority of the students, contrary to previous studies. [26–28] One reason for the observed low prevalence could be the different definitions used to quantify “adequate activity.” Another reason may be due to preparation for the GCE A/L examination, which the Year 13 students were due to sit soon after data collection for this study was undertaken. This exam is highly competitive and thus require them to attend extra classes after school hours, depriving them of additional time for other activities. Both this and a lack of conducive environments could be contributory factors for the inadequacy of overall physical activity. Upper socioeconomic status, male sex, and non-science study streams were the factors associated with an adequate physical activity. The observed gender difference in adequate physical activity was also reported by Singh et al. in New Delhi India. [29] The association with higher socioeconomic status may be attributed to the ability to pay for the use of gymnasiums, combined with the parental use of the same services. [30] Contrasting with the poor knowledge levels among non-science stream students, a significantly higher proportion of these students were engaged in an adequate amount of physical activity.

The low lifetime prevalence of smoking observed among both sexes is consistent with other studies conducted among adolescents in Sri Lanka. [6, 31] However, Katulanda et al. [32] has previously reported a higher prevalence of lifetime smoking in male and female students, of 27%and 13.3% respectively. This higher prevalence may be due to the difference in study settings, as Katulanda et al. [32] conducted the study among students representing the whole of Colombo District, which includes the capital city of Colombo. Maharagama education division, which is situated away from the capital city of Colombo could be considered a semi-urban area, in which the lifestyles of students are less modernized. Likelihood of abstinence from smoking was significant (*p* < 0.001) infinite (certain) among females, which is encouraging to note, given the current trend of the tobacco industry to target more females than males. Another factor that protected adolescents from smoking was having a good knowledge about NCDs. This finding is important from a policy perspective, in that, given the knowledge, the students are more likely to refrain from harmful behaviors. Similar to other studies conducted in Sri Lanka, socio-demographic characteristics did not demonstrate any association with smoking. [31, 33].

The prevalence of those who were current alcohol consumers in the present study was low (11.5%). Similar trends are also observed among teenagers of other Asian countries. [34] In the western countries a higher prevalence among adolescents in comparison to adults is often observed. [34] According to the present study, abstinence from alcohol was significantly higher among female students when their father and/or mother were educated above GCE O/L. The low rates for female drinkers reflect the national trend, where very few females resort to drinking. [35] However, socio-economic status was not observed to be associated with the prevalence of drinking in this study. This fact may also be attributed to the semi-urban nature of the setting. When the parents are better educated, they also guide the children to become more educated and adopt healthy lifestyles and behaviors. However, when parents themselves are less educated, their capacity to advise children is lower, and the children themselves are likely to overrule them.

Concerning smoking and alcohol intake, the social desirability bias was kept to a minimum by making the questionnaires anonymous. As the majority of the respondents were Buddhist, providing information especially on alcohol intake is likely to have been largely distorted, in the absence of anonymity. Its noteworthy that risk behaviours cluster in adolescence, meaning that smokers are more likely to consume alcohol and resort to unhealthy behaviours. Hence; its important that the above are considered when designing programmes. [36, 37].

In the present study, 15% (*n* = 43) of males and 8.1% (*n* = 28) of females were either overweight or obese. The current findings showed a higher prevalence of overweight/obesity compared to a study conducted in the Southern province of Sri Lanka, which reported a prevalence of 4%. [6] This difference could be attributed to the regional differences, and the different cut-off values used to identify overweight and obesity. In the Perera et al. [6] study students whom were ≥25 kg/m^−2^, were considered as overweight or obese, whereas in the present study BMI for age was considered. A significantly higher proportion of females than males had a BMI of less than 25Kgm^−2^. It could be because males have a desire for a heavier physique and muscularity in contrast to females who desire to be thin. [36].

To the best of our knowledge, the current study was the first to determine the level of knowledge on selected NCDs and their associated preventive practices among adolescents in Sri Lanka. As the study was confined to year 13 students of nine schools (consisting of only 41% of all schools in the educational zone), the study findings are generalizable to these schools of Maharagama Education division. However, schools of Maharagama Education division do not differ much from schools of the other educational divisions in Sri Lanka. Further, the confounders considered for the multivariate analysis were confined to factors gathered from students and might have reporting bias; although all possible measures were taken to minimize the reporting bias. Also as with any other study, the potential of unmeasured and residual confounding factors could be a limitation of the study.

As the study participants were students, information related to availability, accessibility, affordability and diversity of diet was not collected, which may have led to the potential for unmeasured or residual confounding. All above are considered as limitations of the study.

## Conclusion and Recommendations

The knowledge and lifestyle practices of adolescent students with regard to NCD’s and their primary prevention were found to be unsatisfactory. This highlights the importance of establishing a system for NCD risk factor surveillance, and implementing awareness raising programs among this group. Conducting regular Global School Health Survey (GSHS) in Sri Lanka is one key recommendation. GSHS is a school-based survey conducted primarily among students aged 13–17 years. Measures should also be taken to incorporate healthy lifestyle education as a major component of the school curriculum, with an aim to provide knowledge as well as a conducive environment for the practice of the same. The strategy for this should include establishment of health promoting schools through school health clubs, with a healthy canteen policy, which is strictly adhered to. The fact that children are considered the best change agents to disseminate knowledge and practice to the family and the community should be maximally explored, as this may prove advantageous for both adolescents and adults equally. [7, 21] However, quick action is imperative if the above objective is to be achieved through children, in order to control the rising epidemic of NCDs.

## Additional files

Additional file 1: The Self Administered Questionnaire to assess Knowledge of non-communicable diseases and practices related to healthy lifestyles among adoloscents. (PDF 7388 kb)

Additional file 2: Table S1. Assignment of score for the component on knowledge. (DOCX 14 kb)

## Abbreviations

BMI: Body mass index
GCE A/L: General Certificate of Education; Advanced-Level
NCDs: Non-communicable diseases
SES: Socio-economic status
WHO: World Health Organization
