# Supplementary material for: Knowledge of non-communicable diseases and practices related to healthy lifestyles among adolescents, in state schools of a selected educational division in Sri Lanka
Source: BMC Public Health. 2017 Jul 26;18:64. doi: 10.1186/s12889-017-4622-z (PMC5530461; doi:10.1186/s12889-017-4622-z)
Supplement: Supplementary file 1 — The Self Administered Questionnaire to assess Knowledge of non-communicable diseases and practices related to healthy lifestyles among adoloscents. (PDF 7388 kb) [file 12889_2017_4622_MOESM1_ESM.pdf]

අනුක්‍රමික අංකය : ....   දිනය : ....

මෙම සමීක්ෂණය සිදු කරන්නේ යෞවනයන්ගේ බෝ නොවන රෝග හා ඒවායේ ප්‍රාථමික සංරක්ෂණ ක්‍රම පිළිබඳ දැනුම හා මෙම රෝග වලින් වැළකීමට ඔබ දරන ප්‍රතිපත්ති පිළිබඳ පරීක්ෂා කිරීමටය.මෙමගින් ලබාගන්නා නිගමනයන් ඔබ වැනිම තුරුණු වියේ පසුවන්නන් සඳහා විවිධ සෞඛ්‍ය, අධ්‍යාපනික වැඩසටහන් නිර්මාණයට යොදා ගැනේ.

මෙම සමීක්ෂණයේදී ඔබ ලබාදෙන තොරතුරුවල රහස්‍යභාවය සුරකින බවට සහතික වෙමි. මෙම ප්‍රශ්න පත්‍රයට පිළිතුරු සැපයීමට හා නොසැපයීම ඔබගේ කැමැත්ත මත සිදුකරන්න. මෙයට පිළිතුරු සැපයුවද නොසැපයුවද එය කිසියම් අයුරකින් ඔබගේ අධ්‍යාපනික කටයුතු සඳහා බලනොපානු ඇත. ප්‍රශ්න පත්‍රයේ ඔබගේ නම නොලියන්න.

ඔබගේ සහයෝගයට ස්තූතියි.

- පිළිතුරු සැපයීමේදී පෑනක් පාවිච්චි කරන්න.
- මෙම ප්‍රශ්න පත්‍රය හොඳින් කියවා පිළිතුරු සපයන්න.
- ප්‍රශ්නය හොඳින් කියවා අදාල කොටුවෙහි (✓) සලකුණු කරන්න.
- කඩ ඉරි යොදා ඇති ස්ථාන මත පිළිතුරු ලියන්න.

1.	ඔබගේ වයස		S1
2.	උපන් දිනය	දිනය	S2
		මාසය	
		වර්ෂය	
3.	ස්ත්‍රී / පුරුෂ භාවය	ස්ත්‍රී	S3
		පුරුෂ	
4.	පාසැලේ නම	....	S4
5.	ඔබගේ ජාතිය	1. සිංහල	S5
		2. දෙමළ	
		3. මුස්ලිම්	
		4. බර්ගර්	S5 Other
		5. වෙනත් (සඳහන් කරන්න.)...	
6.	උසස් පෙළ සඳහා හදාරන විෂය මාලාව	1. විද්‍යාව	S6
		2. ගණිතය	
		3. කලා	
		4. වාණිජ	
7.	a) ඔබගේ පියා දැනට රැකියාවක් කරන්නේද?	1. ඔව් ප්‍රශ්න අංක 7b ට පිළිතුරු සපන්න.	S7
		2. නැත ප්‍රශ්න අංක 8 ට පිළිතුරු සපන්න.	
	b). ඔබගේ පියාගේ රැකියාව	....	S8
8.	ඔබගේ පියාගේ අධ්‍යාපන මට්ටම	1. පාසැල් අධ්‍යාපනය ලබා නොමැත	S9
		2. 1 ශ්‍රේණියේ සිට 5 ශ්‍රේණිය දක්වා	
		3. 6-10 ශ්‍රේණි දක්වා	
		4. අධ්‍යාපන පොදු සහතික පත්‍ර (සා: පෙළ) සමත්	
		5. අධ්‍යාපන පොදු සහතික පත්‍ර (උ: පෙළ) දක්වා	
		6. අධ්‍යාපන පොදු සහතික පත්‍ර (උ: පෙළ) සමත්	
		7. විශ්ව විද්‍යාල අධ්‍යාපනය හෝ ඊට සමාන	
		8. නොදනී	
9.	a) ඔබගේ මව දැනට රැකියාවක් කරන්නේද?	1. ඔව් ප්‍රශ්න අංක 9b ට පිළිතුරු සපයන්න.	S10
		2. නැත ප්‍රශ්න අංක 10 ට පිළිතුරු සපයන්න.	
	b) මවගේ රැකියාව	....	S11

10.	ඔබගේ මවගේ අධ්‍යාපන මට්ටම	1. පාසැල් අධ්‍යාපනය ලබා නොමැත	S12
		2. 1 ශ්‍රේණියේ සිට 5 ශ්‍රේණිය දක්වා	
		3. 6-10 ශ්‍රේණි දක්වා	
		4. අධ්‍යාපන පොදු සහතික පත්‍ර (සා: පෙළ) සමත්	
		5. අධ්‍යාපන පොදු සහතික පත්‍ර (උ: පෙළ) දක්වා	
		6. අධ්‍යාපන පොදු සහතික පත්‍ර (උ: පෙළ) සමත්	
		7. විශ්ව විද්‍යාල අධ්‍යාපනය හෝ ඊට සමාන	
		8. නොදනී	
11.	ඔබගේ නිවසේ ප්‍රධාන පානිය ජල මාර්ගය කුමක්ද?	1. නිවසේ පිහිටා ඇති ආරක්ෂිත ළිඳ	S13
		2. නිවසින් පිටත පිහිටා ඇති ආරක්ෂිත ළිඳ	
		3. ළිඳකින්	
		4. ගෘහස්ථ ජල සැපයුම් පද්ධතිකින්	
		5. මහ පාරේ සවිකර ඇති පොදු ජල මාර්ග පද්ධතියකින්	
		6. ජල බව්සර් මගින් රැගෙන එන ජලය	
12.	ඔබගේ නිවසේ ඇති වැසිකිළි පහසුකම් කුමන වර්ගයට අයත්වේද?	1. ජල මුද්‍රිත වැසිකිළිය	S14
		2. වල වැසිකිළිය	
		3. බාල්දි වැසිකිළිය	
		4. නැත	
13.	ඔබගේ නිවසේ පහත සඳහන් දෑ ඇත්නම් ✓ ලකුණ ද නැතිනම් X ලකුණ ද යොදන්න පිළිතුරු එකකට වඩා ලකුණු කල හැක.	1. විදුලිය	S15 a
		2. ගුවන් විදුලි යන්ත්‍රයක්	S15 b
		3. රූපවාහිනී යන්ත්‍රයක්	S15 c
		4. ශීතකරණය	S15 d
		5. දුරකථනයක්	S15 e
14.	ඔබගේ පවුලේ කිසිම සාමාජිකයෙකුට හෝ පහත සඳහන් වාහන හිමි නම් ✓ ලකුණද නැතිනම් X ලකුණද යොදන්න.	1. බයිසිකලයක්	S16 a
		2. කාර්	S16 b
		3. මෝටර් බයිසිකලයක්	S16 c
		4. වෑන් රථයක්	S16 d
		5. ට්‍රැක්ටරයක්	S16 e
		6. වෙනත් (සඳහන් කරන්න)..	S16 f Other
15.	ඔබගේ නිවසේ පොලොවට යොදා ඇත්තේ මින් කවරක්ද?	1. ටෙරාසෝ / ග්‍රැනයිට් / ටයිල්	S17
		2. සිමෙන්ති	
		2. දැව	
		4. ගොම / මැටි	
		5. වැලි	

02 පුරුදු

ආහාර පුරුදු

16.	ඔබ උදේ ආහාරය වශයෙන් ගෙදරදී පිලියෙල කෙරූ ආහාර භාවිතා කරන සම්භාවිතාව වන්නේ	1. සෑමදිනකදීම	☐	F1
		2. සතියේ දින 5 දී	☐	
		3. සතියේ දින 3 දී	☐	
		4. සති අන්තයේදී	☐	
		5. සතියට තුන්වරක් පමණ	☐	
		6. මසකට වරක් පමණක්	☐	
		7. කිසිවිටක නොමැත	☐	
17.	ඔබ පහත සඳහන් ආහාර වර්ග උදේ ආහාරය වශයෙන් සතියකට දින කීයක් භාවිතා කරනු ලබන්නේද යන්න සඳහන් කරන්න.	1. බත් හා මාළුපිනි	☐	F2 a
		2. හාල් පිටි වලින් සෑදූ ආහාර උදා: ඉදිආප්ප, පිට්ටු, ආප්ප	☐	F2 b
		3. මුං ඇට/ කඩල වැනි දෑ	☐	F2 c
		4. පාන් සමග මාළුපිනි	☐	F2 d
		5. සැන්ඩ්විචස්	☐	F2 e
		6. චයිනිස් රෝල්ස්, පේස්ට්‍රි, කට්ලට්	☐	F2 f
18.	ඔබේ අදහස අනුව පවුලේ අනිකුත් සාමාජිකයින්ට සාපේක්ෂව ඔබට ආහාර සඳහා වැඩියෙන් ලුණු අවශ්‍ය වේද	1. ඔව්, සෑමවිටම	☐	F3
		2. ඔව් සෑමවිටම නොව නමුත් ලුණු මදිවූ විට	☐	
		3. කිසිමවිටෙක නෙමැත	☐	

මීලග ප්‍රශ්න ඔබ සාමාන්‍යයෙන් කෑමට ගන්නා ආහාර හෝ බීම ජාති හා බැඳේ. උදාහරණයක් ලෙස සතියකට වරක් සතියකට දෙවරක්...... මතක තබාගත යුතුයි වැදගත් කරුණ වන්නේ මෙය ඔබ ගන්නා ආහාර පිළිබඳවයි. එබැවින් මෙම ප්‍රශ්න ඔබ ගෙදරදී සහ පිටතදී ගන්නා සියළු ආහාර පිලිබඳ බැඳේ

19.	ඔබ පලතුරු බීම පාවිච්චියට ගන්නා සම්භාවිතාවය වන්නේ (ස්වභාවික)	1. සෑමදිනකදීම	☐	F4
		2. සතියේ දින 5 දී	☐	
		3.සතියට තුන්වරක් පමණ	☐	
		4. සති අන්තයේදී	☐	
		5. මසකට වරක් පමණක්	☐	
		6. කිසිවිටක නොමැත	☐	
20.	ඔබ කෘත්‍රිම බීම වර්ග උදා: කොකා කෝලා, ෆැන්ටා, ක්‍රීම්සෝඩා වැනි බීම භාවිතා කරන සම්භාවිතාව වන්නේ	1. සෑමදිනකදීම	☐	F5
		2. සතියේ දින 5 දී	☐	
		3. සතියට තුන්වරක් පමණ	☐	
		4. සති අන්තයේදී	☐	
		5. මසකට වරක් පමණක්	☐	
		6. කිසිවිටක නොමැත	☐	

21.	ඔබ පලතුරු කෑමට ගන්නා සම්භාවිතාව වන්නේ සතියකට කී වරක්ද	1. සෑමදිනකදීම	F6
		2. සතියේ දින 5 දී	
		3. සතියට තුන්වරක් පමණ	
		4. සතියකට දෙවරක් පමණි	
		5. මසකට වරක් පමණක්	
		6.කිසිවිටක නොමැත	
22.	ඔබ සාමාන්‍යයෙන් එලවළු හා පලාවර්ග බෙදීම් (Serving) කොපමණ දිනකට අනුබව කරන්නේද? එක් බෙදීමක් යනු එලවළු හෝ පලා වර්ග මේස හැඳි 02 කි උදා- ඔබ දිවා ආහාරය සඳහා එලවළු ජාති 02ක් ආහාරයට ගත්තේ නම් එය එලවළු බෙදීම් (Serving) 02 ක් ලෙස සැලකේ. එලෙස ඔබ එක් දිනයක් තුළදී ආහාරයට ගන්නා එලවළු හා පලා බෙදීම් ගණන	1. ඔබ දිනක ආහාරයට ගන්නා එලවළු බෙදීම් ගණන	F7 a
		2. ඔබ දිනකට ආහාරයට ගන්නා පලා බෙදීම් ගණන	F7 b

ව්‍යායාම් පිලිබදව

23.	ශාරීරික ව්‍යායාම් ඔබගේ සෞඛයට යෝග්‍ය යැයි ඔබ සිතනවාද	1. ඔව්	E1
		2. නැත	
		3. නොදනී	
24.	ඔබ කිසියම් හො ශාරීරික/ ව්‍යායාම ව්‍යායාමයක යෙදෙනවාද?	1. ඔව් ප්‍රශ්න අංක 25 ට යන්න	E2
		2. නැත ප්‍රශ්න අංක 28 ට යන්න	
25.	ඔබ යෙදෙන්නා වු ශාරීරික ව්‍යායාම වන්නේ (එකක් හො කිපයක් ලකුණු කල හැක)	1. ක්‍රීඩා - උදා ක්‍රිකට් පිහිනීම් නෙට්බෝල්	E3
		2. ස්වවායු ව්‍යායාම් (එරොබික්ස්)	
		3. යෝග ව්‍යායාම්	
		4. නැටුම්	
		5. වෙනත් (සඳහන් කරන්න)...	
26.	ඔබ ව්‍යායාම් කරන දිනයන්හි ඒ සඳහා ගත කරන කාල වේලාව වන්නේ	1. < මිනිත්තතු 30	E4
		2. මිනිත්තු 30	
		3. < මිනිත්තතු 45	
		4. මිනිත්තතු 45	
		5. පැය යි	
		6. නිසැකව කිව නොහැක	
		7.වෙනත් (සඳහන් කරන්න)............................	
27.	ඔබ ඉහත සඳහන් ව්‍යායාමවල සතියකට දින කීයක් යෙදෙනවාද?	1. සතියකට දින	E5
		2. කිසිවක් කිව නොහැක	

දුම්පානය පිළිබඳ

මෙම දිනවල ලාබාල අවධියේ සිට පුද්ගලයන් දුම්පානය කරනු ලැබේ. ස්ත්‍රී පුරුෂ දෙපාර්ශවයම මේ ක්‍රියාවලි වල යෙදෙන බව අධ්‍යයනයන්ගෙන් සොයාගෙන ඇත. මිත්‍රයන්ගේ බලපෑම හානිකර ප්‍රතිඵල පිළිබඳ දැනුම නැතිවීම හා කුතුහලය මේවායේ නිරත වීම හේතු කිහිපයකි.

28.	ඔබ කිසිදිනක දුම්පානය කර තිබේද?	1. ඔව් ☐ ප්‍රශ්න අංක 32 ට යන්න.	T1
		2. නැත ☐ ප්‍රශ්න අංක 37 ට යන්න	
29.	ඔබ දැනට දුම්පානය කරන්නේද?	1. ඔව් ☐ ප්‍රශ්න අංක 33 ට යන්න	T2
		2. නැත ☐ ප්‍රශ්න අංක 35 ට යන්න	
30.	ඔබ දුම්පානය කරන වාර ගණන කුමක්ද?	1. දිනපතා ☐	T3
		2. සතියේ දින 5 දී ☐	
		3. සතියකට දින 3ක් පමණ ☐	
		4. සති අන්තයේදී ☐	
		5. මසකට සැරයක් පමණකි ☐	
		6. උත්සව අවස්ථාවකදී පමණක් ☐	
		7. ඉඳහිට ☐	
31.	ඔබ දිනකට සිගරට් කීයක් පානය කරන්නේද?	....	T4
32.	ඔබ පළමු වරට දුම්පානය කරන විට ඔබගේ වයස	1. වයස ☐	T5
		2. මතක නැත ☐	

මධ්‍යසාර භාවිතය

මෙම දිනවල ලාබාල අවධියේ සිට පුද්ගලයන් මධ්‍යසාර භාවිතා කරනු ලැබේ. ස්ත්‍රී පුරුෂ දෙපාර්ශවයම මේ ක්‍රියාවලි වල යෙදෙන බව අධ්‍යයනයන්ගෙන් සොයාගෙන ඇත. මිත්‍රයන්ගේ බලපෑම හානිකර ප්‍රතිඵල පිළිබඳ දැනුම නැතිවීම හා කුතුහලය මේවාගේ නිරත වීම හේතු කිහිපයකි.

33.	ඔබ කිසිදිනක හෝ මධ්‍යසාර භාවිත කර තිබේද ?	1. ඔව් ☐ ප්‍රශ්න අංක 38	A1
		2. නැත ☐ ප්‍රශ්න අංක 43 (බෝ නොවන රෝග)	
34.	ඔබ දැනට මධ්‍යසාර භාවිතා කරන්නේද ?	1. ඔව් (දිනපතා) ☐ ප්‍රශ්න අංක 39 ට යන්න	A2
		2. නැත ☐ ප්‍රශ්න අංක 41 ට යන්න	
35.	ඔබ මධ්‍යසාර භාවිතා කරන වාර ගණන වන්නේ?	1. දිනපතා ☐	A3
		2. සතියේ දින 5 දී ☐	
		3. සතියකට දින 3ක් පමණ ☐	
		4. සති අන්තයේදී ☐	
		5. මසකට සැරයක් පමණක් ☐	
		6. උත්සව අවස්ථාවකදී පමණක් ☐	
		7. ඉඳහිට ☐	

36.	ඔබ සාමාන්‍යයෙන් භාවිතා කරන මධ්‍යසාර වර්ගය හා දිනකට භාවිතා කරන මධසාර ප්‍රමාණය සඳහන් කරන්න.

උදා බියර් ✓ 750ml බෝතල් 1\2

මධ්‍යසාර වර්ගය අදාල	පිලිතුර ලකුණු කරන්න	වරකට ගන්නා ප්‍රමාණය 750ml බොතල් වලින් සඳහන් කරන්න	
1. බියර්			A4 a
2. වයින්			A4 b
3. අරක්කු			A4 c
4. ජින්			A4 d
5. විස්කි			A4 e
6. බ්‍රැන්ඩි			A4 f

37.	ඔබ පලමුවරට මධ්‍යසාර භාවිතා කරන විට ඔබගේ වයස වුයේ?	1. වයස ☐	A5
		2. මතක නැත ☐	

03 බෝ නොවන රෝග පිලිබද දැනුම

පහත සඳහන් ප්‍රතිචාර වලට නිවැරදි ප්‍රතිචාර ඉදිරියෙන් ලකුණද වැරදි ප්‍රතිචාර සඳහා නැත කොටුව ලකුණද නොදනී නම් නොදනී කොටුව ලකුණද යොදන්න. (එක් පිලිතුරකට වඩා සටහන් කලහැක)

ඔව් / නැත / නොදනී

			ඔව් / නැත / නොදනී	
38.	පහත සඳහන් රෝග අතරින් **බෝ නොවන** රෝග ලෙස සැලකිය හැක්කේ කවර රෝගය/රෝගය ද?	1. දියවැඩියාව	1 2 3	N1 a
		2. හෘද ආබාධ	1 2 3	N1 b
		3. අංශභාගය	1 2 3	N1 c
		4. ශ්වසන පද්ධතියේ නිදන් ගත රෝග	1 2 3	N1 d
		5. ක්ෂය රෝගය	1 2 3	N1 e
		6. පිළිකාව	1 2 3	N1 f
39.	පහත සඳහන් කවරක් **බෝ නොවන** රෝග සඳහා හේතු සාධක වන්නේද?	1. දුම්බීම	1 2 3	N2 a
		2. අධික ලෙස මධ්‍යසාර භාවිතය	1 2 3	N2 b
		3. අහිතකර ආහාර පුරුදු	1 2 3	N2 c
		4. ශරීරික ව්‍යායාම නොමැතිකම	1 2 3	N2 d
		5. උණු කර නිවාගත් ජලය පානය නොකිරී	1 2 3	N2 e

දියවැඩියාව

			ඔව් / නැත / නොදනී	
40.	පහත සඳහන් කවරක් දියවැඩියාව වැලදීමේ අවධානම වැඩි කරනු ලබයිද?	1. ශාරීරික ව්‍යායාම නොමැති කම	1 2 3	N3 a
		2. මධ්‍යසාර භාවිතය	1 2 3	N3 a
		3. ආරය	1 2 3	N3 c
		4. අධික තරබාරුව	1 2 3	N3 d
		5. අහිතකර ආහාර පුරුදු	1 2 3	N3 e

ඔව් / නැත / නොදනී

අංකය	ප්‍රශ්නය	පිළිතුර	ඔව්	නැත	නොදනී	කේතය
41.	දියවැඩියාවේ රෝග ලක්ෂණ වන්නේ පහත සඳහන් ලක්ෂණ අතරින් කවරක්ද?	I. අධික පිපාසය	1	2	3	N4 a
		2. රාත්‍රී කාලයේදී නිතර නිතර මුත්‍රා කිරීමේ අවශ්‍යතාවය	1	2	3	N4 b
		3. පෙනීමේ දුර්වලතා	1	2	3	N4 c
		4. තුවාල සුව වීමේ ප්‍රමාදය	1	2	3	N4 d
		5. නිතර නිතර ලෙඩ රෝග වලට භාජනය වීම	1	2	3	N4 e
		6. දීර්ඝ කාලීනව පවතින කැස්ස	1	2	3	N4 f
		7. කැස්ස සමග ලේ පිටවීම	1	2	3	N4 g
		8. බර අඩුවීම	1	2	3	N4 h
42.	දියවැඩියාව මගින් හානි සිදුවන ඉන්ද්‍රියය / ඉන්ද්‍රියයන් වන්නේ	1. ඇස්	1	2	3	N5 a
		2. වකුගඩු	1	2	3	N5 b
		3. හෘදය	1	2	3	N5 c
		4. පර්යන්ත ස්නායු	1	2	3	N5 d
		5. අක්මාව	1	2	3	N5 e
		6. අග්න්‍යාසය	1	2	3	N5 f
43.	දියවැඩියාව වැළඳීමෙන් ඇතිවන අනිටු ඵල කවරේද?	1. මරණය	1	2	3	N6 a
		2. ශරීරයේ විවිධ ඉන්ද්‍රියයන්ට හානි පැමිනිවීම	1	2	3	N6 b
		3. අතපය හිරිගතිය නිසා කාර්යයන් කරගැනීමේ අපහසුව	1	2	3	N6 c
		4. දීර්ඝ කාලින ප්‍රතිකාර ලබාගතයුතු රෝගියෙකු බවට පත්වීම	1	2	3	N6 d
		5. අස්ථි විකෘතිවීම නිසා ඇවිදීමේ අපහසුවතාවය	1	2	3	N6 e
44.	දියවැඩියාව වැළඳීමෙන් ඈත් විය හැක්කේ කුමන සෞඛ්‍යම පුරුදු පිළිපැදීමෙන්ද?	1. සම බල ආහාර වේලක් අනුභව කිරීමෙන්	1	2	3	N7 a
		2. පිෂ්ඨය බහුල ආහාර වලින් වැළකී සිටීමෙන්	1	2	3	N7 b
		3. අධික සීනි භාවිතයෙන් ඈත් වීමෙන්	1	2	3	N7 c
		4. උසට සරිලන බරක් පවත්වා ගැනීමෙන්	1	2	3	N7 d
		5. මධ්‍යසාර භාවිතය නතර කිරීමෙන්	1	2	3	N7 e
		6. අධික ලුණු සහිත ආහාර සීමා කිරීම	1	2	3	N7 f
		7. දියවැඩියාව සඳහා ලබාගන්නා ප්‍රතිකාර නිසිඅයුරින් පිළිපැදීමෙන්	1	2	3	N7 g

අධි රුධිර පීඩනය

ඔව් / නැත / නොදනී

අංකය	ප්‍රශ්නය	පිළිතුර	ඔව්	නැත	නොදනී	කේතය
45.	අධි රුධිර පීඩනය වැලදීමේ අවධානම වැඩිකරනු ලබන සාධක කවරක්ද ?	1. ආරය	1	2	3	N8 a
		2. අධික තරබාරුව	1	2	3	N8 b
		3. අධික මත්පැන් භාවිතය	1	2	3	N8 c
		4. අධික ලුණු භාවිතය	1	2	3	N8 d
		5. සමහර ඖෂධ භාවිතය	1	2	3	N8 e
		6. ඉහත සඳහන් කිසිවක් නොමැත	1	2	3	N8 f

			ඔව් / නැත / නොදනී	
46.	අධි රුධිර පීඩනය වැළඳී ඇති රෝගියෙකුගේ තිබිය හැකි රෝග ලක්ෂණ මේවා අතරින් කවරකද? (එක පිලිතුරකට වඩා සඳහන් කලහැක)	1. කිසිදු රෝග ලක්‍ෂණයක් නොමැත	1 2 3	N9 a
		2. අධික හිසරදය	1 2 3	N9 b
		3. කරකැවිල්ල	1 2 3	N9 c
		4. පෙනීමේ දුර්වලතා	1 2 3	N9 d
		5. ඔක්කාරය/ වමනය	1 2 3	N9 e
		6. තුවාල සුව වීමට කල් ගත වීම	1 2 3	N9 f
47.	අධි රුධිර පීඩනයට ප්‍රතිකාර ලබා නොගැනීම නිසා එමගින් හානි සිදුවන ඉන්ද්‍රියන් වනුයේ?	1. වකුගඩු	1 2 3	N10 a
		2. හෘදය	1 2 3	N10 c
		3. මොලය	1 2 3	N10 d
		4. අක්මාව	1 2 3	N10 e
		5. ආමාශය	1 2 3	N10 f
48.	අධි රුධිර පීඩනය වැළදීම වලක්වා ගත හැකි ක්‍රම වන්නේ?	1. සිරුරේ බර පාලනය	1 2 3	N11 a
		2. එන්නත් දීම මගින්	1 2 3	N11 b
		3. මධ්‍යසාර භාවිතයෙන් වැළකී සිටීම	1 2 3	N11 c
		4. ලුණු භාවිතය සීමා කිරීම	1 2 3	N11 d
		5. දිනපතා ශාරීරික ව්‍යායාම වල යෙදීම	1 2 3	N11 e
		6. මානසික ආතතිය දුරු කිරීම	1 2 3	N11 g
		7. දියවැඩියාව / කොලෙස්ටරෝල් වැනි රෝගයන්ට ප්‍රතිකාර ලබාගැනීම මගින්	1 2 3	N11 h

හෘදයාබාධ

			ඔව්/ නැත / නොදනී	
49.	හෘදආබාධයක් යනු කුමක්ද?	1. ඔක්සිජන් හා පෝෂ්‍ය ද්‍රව්‍ය සැපයුම අඩුවී රුධිර සැපයුම අඩාල වී (නාල කුඩා වී / අවහිර වීම නිසා) හෘදපේශියට සදාකාලිකව හානි සිදුවීම	1 2 3	N12 a
		2. පර්ශු කුඩුවට හානි සිදුවීම	1 2 3	N12 b
		3. හුස්මගැනීමේ වේගය වැඩිවීම	1 2 3	N12 c
		4. හෘද ස්පන්දන වේගය වැඩිවීම	1 2 3	N12 d
50.	හෘදයාබාධයක් හඳුනා ගැනීමේ රෝග ලක්ෂණ කවරේද?	1.විවේකීව සිටීමේදී හට ගන්නා පපුවේ වේදනාව	1 2 3	N13 a
		2. වම් අත දිගේ හා බෙල්ලේ වම් පැත්තේ පැතිර යන පපුවේ වේදනාව	1 2 3	N13 b
		3. පපුවේ වේදනාව සමග අධික දහඩිය දැමීම හා වමනය	1 2 3	N13 c
		4. පපුවේ වේදනාව සමග හුස්ම ගැනීමේ අපහසුතාවය	1 2 3	N13 d
51.	පහත සඳහන් කවරක් හෘදයාබාධ සෑදීමේ අවධානම වැඩි කිරීමට හේතුවේද?	1. රුධිරයේ කොලෙස්ටරෝල් වැඩිවීම	1 2 3	N14 a
		2. දුම්පානය	1 2 3	N14 b
		3. දියවැඩියාව	1 2 3	N14 c
		4. අධික තරබාරුව	1 2 3	N14d
		5. අධි රුධිර පීඩනය	1 2 3	N14 e

			ඔව්	නැත	නොදනී	
51.	හෘදයාබාධ වැලදීමෙන් සිදුවන අතුරු ආබාධ කවරේද?	1. මරණය	1	2	3	N15 a
		2. එදිනෙදා වැඩ කටයුතු කර ගැනීමේ අපහසුතාවය	1	2	3	N15 b
		3. නැවත හෘදයාබාධයක් වැළදීමේ අවධානම	1	2	3	N15 c
		4. අතුරු ආබාධ කිසිවක් නැත	1	2	3	N15 d
52.	ඔබට හෘදයාබාධ සෑදීමෙන් වැලකී සිටීමට පිලිපැදිය යුතු වන්නේ කවර උපදෙස් / උපදෙසක් ද?	1. අධික මේද සහිත ආහාර වලින් වැලකී සිටීම	1	2	3	N16 a
		2. නිතිපතා ශාරීරික ව්‍යායාම වල යෙදීම	1	2	3	N16 b
		3. දුම්පානය නතර කිරීම	1	2	3	N16 c
		4. ශරීරය උසට සරිලන බරකින් යුතුව තබාගැනීම	1	2	3	N16 d
		5. කෑමට පෙර අත සබන් දමා යෙදීමෙන්	1	2	3	N16 e
		6. සමබල පෝෂ්‍යදායී ආහාර වේලක් භාවිතය	1	2	3	N16 f

අංශභාගය			ඔව්	නැත	නොදනී	
53.	ආඝාතය (අංශභාගය යනු කුමක්ද)	1. රුධිර සැපයුම බාධාවක් නිසා මොළයට ඔක්සිජන් හා පෝෂණ ද්‍රව්‍ය සැපයීමේ ඌනතාවය නිසා මොළයේ සෛලවලට හානිසිදුවීම	1	2	3	N17 a
		2. සුෂුම්නාවට හානි සිදුවීම නිසා ඇතිවන රෝගී තත්ත්වයකි	1	2	3	N17 b
		3. හිස් කබල තුවාල වීම	1	2	3	N17 c
53.	අංශභාගය හඳුනා ගැනීමේ රෝග ලක්ෂණ කවරේද?	I. එකවර ඇතිවූ ගොත ගැසීම	1	2	3	N18 a
		2. හදිසියේ ඇතිවූ ශරීරයේ එක් අංශයක අප්‍රාණික බව	1	2	3	N18 b
		3. හදිසියේ ඇතිවූ ශරීරයේ එක් අංශයක හැඟීම් දැනීම් නොමැති වීම	1	2	3	N18 c
		4. පෙනීමේ විශමතා	1	2	3	N18 d
54.	පහත සඳහන් කවරක් අංශභාගය සෑදීමේ අවධානම වැඩි කිරීමට හේතුවේද?	1. රුධිරයේ කොලොස්ටරෝල් වැඩිවීම	1	2	3	N19a
		2. දුම්පානය	1	2	3	N19b
		3. දියවැඩියාව	1	2	3	N19c
		4. අධික තරබාරුව	1	2	3	N19d
		5. අධි රුධිර පීඩනය	1	2	3	N19e
55.	අංශභාගය (ආඝාතය) සෑදීමෙන් ඇතිවන අතුරු ඵල කවරෙක්ද?	1. මරණය	1	2	3	N20a
		2. ශරීරයේ අංශයක අප්‍රානික බව	1	2	3	N20b
		3. කථනයේ අපහසුතා	1	2	3	N20c
		5. එදිනෙදා කාර්ය කර ගැනීමේ අපහසුතාවය	1	2	3	N20d
		5. අතුරු ආබාධ කිසිවක් නැත	1	2	3	N20e

			ඔව්	නැත	නොදනී	
56.	ඔබ ආඝාතය සෑදීමේ අවධානමෙන් වැලකීමට පිලිපැදිය යුතු උපදෙස් කවරේද?	1. අධික මේද සහිත ආහාර වලින් වැලකී සිටීම	1	2	3	N21 a
		2. නිතිපතා ශාරීරික ව්‍යායාම වල යෙදීම	1	2	3	N21 b
		3. දුම්පානය නතර කිරීම	1	2	3	N21 c
		4. ශරීරය උසට සරිලන බරකින් යුතුව තබාගැනීම	1	2	3	N21 d
		5. කෑමට පෙර අත සබන් දමා යෙදීමෙන්	1	2	3	N21 e
		6. සමබල පෝෂ්‍යදායී ආහාර වේලක් භාවිතය	1	2	3	N21 f

පිළිකාව

			ඔව්	නැත	නොදනී	
57.	පිළිකාවක් හඳුනා ගැනීමේදී පලමු අනතුරු හැගවීමේ සංඥා වන්නේ?	1. ශරීරයේ මොනයම් හෝ ස්ථානයක වේදනාවක් රහිත ගැටිත්තක් තිබීම	1	2	3	N22 a
		2. දීර්ඝ කාලීනව සුව නොවන තුවාලයක්	1	2	3	N22 b
		3. දීර්ඝ කාලීනව පවතින සුව නොවන කැස්සක්	1	2	3	N22 c
		4. මල සමග ලේ සිදම් පිටවීම	1	2	3	N22 d
		5. ගිලීමේ අපහසුතාවයක්	1	2	3	N22 e
		6. හේතුවක් රහිතව ශරීරයේ බර අඩු වීම	1	2	3	N22 f
		7. වැසිකිලියාමේ වෙනසක්	1	2	3	N22 g
		8. කට හඬේ සිදුවන වෙනසක්	1	2	3	N22 i
		9. වේදනා සහිත ගැටිත්තක් හෝ ගෙඩියක්	1	2	3	N22 j
		10. කැස්ස සමග රුධිරය පිටවීම	1	2	3	N22 k
57.	ඔබ පිලිකාව යන රෝගී තත්ත්වය පිලිබඳ දැනුවත්ව තිබිය යුත්තේ ඇයි?	1. එය මරණය ලගාකරවන රෝගයක් නිසා	1	2	3	N23 a
		2. එය වැලඳුනහොත් එදිනෙදා කාර්යයන් කිරීමට අපහසුවන නිසා	1	2	3	N23 b
		3. එය වැලඳුනහොත් දීර්ඝකාලීන ප්‍රතිකාර ලබාගැනීමට සිදුවන නිසා	1	2	3	N23 c
		4. එය 100 % සුවකල හැකි රෝගී තත්ත්වයක් නිසා	1	2	3	N23 d
		5. අධික ලෙස දුක්පීඩා විඳීමට සිදුවීම නිසා	1	2	3	N23 e
58.	බොහෝ පිළිකා සෑදීමට හේතු සාධකය /සාධක වන්නේ මෙයින් කවරේද?	1. දුම්බීම	1	2	3	N24 a
		2. දුම්කොල හා පුවක් සමග බුලත් විට කෑම	1	2	3	N24 b
		3. ආහාර වලට යොදන විවිධ රසකාරක කල් තබා ගැනීමේ ද්‍රව්‍ය ආදිය	1	2	3	N24 c
		4. පිලිස්සු මස් / මාළු (BBQ) වැනි ආහාර නිතර ගැනීම	1	2	3	N24 d
		5. දීර්ඝ කාලීනව පාරජම්බුල කිරණ වල (හිරු එළියට) නිරාවරණය	1	2	3	N24 e
		6. අධික තරබාරුව	1	2	3	N24 d
		7. බහු පුරුෂ සේවනය	1	2	3	N24 e
		8. කාබෝහයිඩ්‍රේට් අධික, මේද අධික හා ප්‍රෝටීන ප්‍රමාණයෙන් අවම ආහාර අනුභවය	1	2	3	N24 f
		9. පළතුරු හා එළවළු පරිභෝජනයේ මදකම	1	2	3	N24 g

ඔව් / නැත / නොදනී

59.	පිළිකාවක් සෑදීමේ අවධානම අඩු කර ගැනීමට පිළිපැදිය හැකි මාර්ග වන්නේ ?	1. දුම්බීමෙන් නැවැත්වීම	1	2	3	N25 a
		2. බුලත්විට කෑම නැවැත්වීම	1	2	3	N25 b
		3. එලවළු හා පළතුරු බහුලව අනුභවය කිරීම	1	2	3	N25 c
		5. ස්වභාවික ආහාර හැකි සෑමවිටම පරිභෝජනය	1	2	3	N25 d
		5. නිතිපතා ව්‍යායාමවල යෙදීම	1	2	3	N25 e
		6. දීර්ගකාලීනව හිරු එළියට නිරාවරනය/ බඳුන් නොවීම	1	2	3	N25 f
		7. එක් ලිංගික සහකරුවෙකු / සහකාරියක් පමණක් තෝරා ගැනීම	1	2	3	N25 g
60.	බෝ නොවන රෝග පිළිබඳ දැනුම සහ ඒවායේ ප්‍රාථමික සංරක්ෂණ ක්‍රම පිළිබඳ දැනුම අපට වැදගත් වන්නේ ඇයි?	1. බෝ නොවන රෝග වැලදීමෙන් ආරක්ෂා වීමට	1	2	3	N26 a
		2. බෝ නොවන රෝග වැලදී ඇත්නම් රෝගය පාලනය කිරීමට	1	2	3	N26 b
		3. පවුලේ සාමාජිකයන් බෝ නොවන රෝග වලින් ආරක්ෂා කිරීමට	1	2	3	N26 c

04 පෝෂණය සම්බන්ධ දැනුම

61.	සමබල ආහාර වේලක අඩංගු ආහාර සංඝටක කවරේද?	1. පිෂ්ඨය	1	2	3	K1 a
		2. ප්‍රෝටීන	1	2	3	K1 b
		3. මේදය	1	2	3	K1 c
		4. විටමින්	1	2	3	K1 d
		5. ඛණිජලවණ	1	2	3	K1 e
62	ඔබගේ ආහාර වේල පෝෂ්‍යජනක කර ගැනීමට ඔබ අනුගමනය කර හැකි ක්‍රියා මාර්ග වන්නේ	I. දිනපතා ආහාර වේල සඳහා එලවළු පළතුරු එකතු කර ගැනීම	1	2	3	K2 a
		2. ලුණු අධික ආහාර ගැනීමෙන් වැලකී සිටීම උදා-පීට්සා චිස්-අල බැදුම්(French fries)	1	2	3	K2 b
		3. තන්තු සහිත ආහාර වැඩි වශයෙන් අනුභවය කිරීම උදා-පළතුරුල එළවළු	1	2	3	K2 c
		4. ඔමේගා- 3 බහුල කුඩා මාළු නිතර ආහාරයට එක් කර ගැනීම උදා - සැමන්	1	2	3	K2 d
		5. මේද අධික ආහාර සීමා කිරීම	1	2	3	K2 e
		6. ආහාර වේල් ගැනීම අතපසු කිරීම (diet)	1	2	3	K2 f
63.	පහත සඳහන් ආහාර අතරින් ප්‍රෝටීන් බහුල ආහාර වනුයේ කවරක්ද?	1. මාළු උදා කුඩා මාළු, කෙලවල්ලා	1	2	3	K3 a
		2. මස් වර්ග	1	2	3	K3 b
		3. බිත්තර සුදු මදය	1	2	3	K3 c
		4. ධාන්‍ය හා පියලි වර්ග වල	1	2	3	K3 d
		5. පලතුරු හා පලා වර්ග වල	1	2	3	K3 e
64.	පහත සඳහන් ආහාර වර්ග වලින් පිෂ්ඨය බහුල ආහාර වර්ග කවරේද?	I. බත්, හාල්පිටි යොදා පිසූ ආහාර	1	2	3	K4 a
		2. අල, බතල වැනි අල වර්ග	1	2	3	K4 b
		3. කෝස් දෙල්	1	2	3	K4 c
		5. කිරිවලින් තැනූ ආහාර උදා: යෝගට්, චීස්,	1	2	3	K4 d
		5. චොකලට්	1	2	3	K4 e

			ඔව් / නැත / නොදනී	
65.	ක්ෂණික ආහාර (Junk food, fast food) අධි කැලරි ප්‍රමාණයකින් යුක්ත නමුත් ඒවාගේ පෝෂණ ගුණය ඉතා අඩුය. පහත සඳහන් ආහාර අතරින් ක්ෂණික ආහාර වන්නේ කවරක්ද?	1. අල බැදුම් (Potato chip)	1 2 3	K5 a
		2. ටොෆි	1 2 3	K5 b
		3. චුයින්ගම් (chewing gum)	1 2 3	K5 c
		4. බර්ගර් වැනි ආහාර (burgers)	1 2 3	K5 d
		5. පීට්සා (Pizza)	1 2 3	K5 e
		6. පලතුරු වර්ග.	1 2 3	K5 f
66.	ක්ෂණික ආහාර නිතර ගැනීමෙන් සිදුවන අහිතකර ප්‍රතිඵල කවරක්ද?	1. දියවැඩියාව සෑදීමේ ප්‍රවනතාව වැඩිවීම	1 2 3	K6 a
		2. අධික තරබාරුව	1 2 3	K6 b
		3. දත් දිරායාම	1 2 3	K6 c
		4. හෘද රෝග සෑදීමේ අවධානම වැඩිවීම	1 2 3	K6 d
67.	පලතුරු සහ එලවළු අනුභවය වැදගත් වන්නේ ඇයි?	1. පලතුරු සහ එලවළු විටමින් සහ ඛනිජලවණ වලින් පොහොසත් නිසා	1 2 3	K7 a
		2. තන්තු ප්‍රභවයක් බැවින්	1 2 3	K7 b
		3. සමහර එලවළු ප්‍රෝටීන ප්‍රභවයක් නිසා	1 2 3	K7 c
		4. තරබාරුව අඩු කිරීමට සමත් නිසා	1 2 3	K7 d
		5. දියවැඩියාව, අධිරුධිර පීඩනය, හෘදයාබාධ සෑදීමේ අවධානම අඩුකරන නිසා	1 2 3	K7 e
		6. පිළිකා සෑදීමේ අවධානම අඩු කරන නිසා	1 2 3	K7 f

05 ශාරීරික ව්‍යායාම පිළිබඳ දැනුම

			ඔව් / නැත / නොදනී	
68.	අධික තරබාරුව ඔබගේ ශරීර සෞඛ්‍යයට බලපාන්නේ කෙසේද?	1. එය දියවැඩියාව සෑදීමේ ප්‍රවනතාවය වැඩි කරයි	1 2 3	K8 a
		2. එය අධිරුධිර පීඩනය සෑදීමේ ප්‍රවනතාවය වැඩි කරයි	1 2 3	K8 b
		3. එය හෘද ආබාධ/ අංශ භාගය සෑදීමේ ප්‍රවනතාවය වැඩි කරයි	1 2 3	K8 c
		4. එය සන්ධි ආබාධ උදා - ආතරයිටිස් සෑදීමේ ප්‍රවනතාව වැඩි කරයි	1 2 3	K8 d
69.	ශාරීරික ව්‍යායාම කිරීමෙන් බෝ නොවන රෝග සෑදීමේ ප්‍රවනතාව අඩු කරන්නේ කෙසේද?	1. රුධිරයේ සීනි අඩු කිරීමෙන්	1 2 3	K9 a
		2. රුධිරයේ රුධිර ලිපිඩ අඩු කිරීම මගින්	1 2 3	K9 b
		3. අධි රුධිර පීඩනය ඇත්නම් පීඩනය අඩු කිරීම මගින්	1 2 3	K9 c
		4. රුධිර සංසරණ පද්ධතියේ මනා ක්‍රියාකාරීත්වය පවත්වා ගැනීමෙන්	1 2 3	K9 d
		5. මානසික ආතතිය වැඩි කිරීම මගින්	1 2 3	K9 e
		6. අස්ථි හා මාංශ පේශීන් සවිමත් කිරීම මගින්	1 2 3	K9 f
		7. ශරීරයේ බර අඩු කිරීම මගින්	1 2 3	K9 g

දුම්පානය

ඔව් / නැත / නොදනී

70.	දුම්පානය ශරීරයට අහිතකර වේග දුම්පානය මගින් හානි සිදුවිය හැකි ඉන්ද්‍රියන් වන්නේ පහත සඳහන් කවරක්ද?	1. හෘදය	1 2 3	K10 a
		2. ආමාශය	1 2 3	K10 b
		3. රුධිරවාහිනී	1 2 3	K10 c
		4. පෙනහළු	1 2 3	K10 d
		5. අක්මාව	1 2 3	K10 e
71.	දුම්පානය නිසා වැළඳිය හැකි රෝග ආබාධ කවරේද?	1. පිළිකා	1 2 3	K11 a
		2. හෘද ආබාධ	1 2 3	K11 b
		3. අංශභාගය	1 2 3	K11 c
		4. ශ්වසන පද්ධතියේ නිදන්ගත රෝග	1 2 3	K11 d
		5. අක්මාවේ ආසාදන (හෙපටයිටිස්)	1 2 3	K11 e

මධ්‍යසාරය

72.	මධ්‍යසාර භාවිතය ශරීරයට අගුණ වේ. මධ්‍යසාර භාවිතය නිසා හානි සිදුවිය හැකි ඉන්ද්‍රියයන් කවරක්ද?	1. අක්මාව		K12 a
		2. ආමාශය		K12 b
		3. අග්න්‍යාශය		K12 c
		4. මොළය		K12 d
		5. හෘදය		K12 e
		6. ස්නායු		K12 f
73.	මධ්‍යසාර භාවිතය නිසා වැළඳිය හැකි රෝගාබාධ කවරක්ද?	1. බඩේ දැවිල්ල (ගැස්ට්‍රයිටිස්)		K13 a
		2. ආමාශගත තුවාල		K13 b
		3. අක්මාවේ සිරෝසිස් තත්ත්වය		K13 c
		4. අග්න්‍යාශයික ප්‍රදාහය		K13 d
		5. හෘදයාබාධ		K13 e
		6. මානසික රෝගී තත්ත්වයන්		K13 f

06 බෝ නොවන රෝග හා ඒවායේ ප්‍රාථමික සංරක්ෂණ ක්‍රම පිලිබඳව දැනුම ලබාගත් මාධ්‍යය

74.	ඉහත රෝගයන් හා ඒවායේ ප්‍රාථමික සංරක්ෂණ ක්‍රම පිළිබඳව දැනුම ලැබූ මාධ්‍ය වන්නේ (ප්‍රමුඛතාව මත 1 2 3 4 5 6 7 ආදිය යොදන්න)	I. පාසැල		M1 a
		2. දෙමාපියන්		M1 b
		3. යහළුවන්		M1 c
		4. රූපවාහිනිය		M1 d
		5. ගුවන් විදුලිය		M1 e
		6. සෞඛ්‍ය ක්ෂේත්‍රයේ රාජකාරී නියතුවන (වෛද්‍යවරුන් / හෙදනිලධාරීන්)		M1 f
		7. පුවත්පත් සඟරා මගින්		M1 g
		8. වෙනත් (සඳහන් කරන්න)		M1 Other

75.	බෝ නොවන රෝග පිළිබඳ දැනුම ඔබ ලබා ගැනීමට කැමති මාධ්‍යය වන්නේ (ප්‍රමුඛතාව මත 1 2 3 4 5 6 7 ආදිය යොදන්න)	1. පාසැල	M2 a
		2. දෙමාපියන්	M2 b
		3. යහළුවන්	M2 c
		4. රූපවාණිය	M2 d
		5. ගුවන් විදුලිය	M2 e
		6. සෞඛ්‍ය සේවයේ නියතුවුවන් (උදා- වෛද්‍යවරුන්/ හෙදියන්)	M2 f
		7. පුවත්පත් සඟරා මඟින්	M2 g
		8. වෙනත් (සඳහන් කරන්න)	M2 Other
