# Supplementary material for: Knowledge of non-communicable diseases and practices related to healthy lifestyles among adolescents, in state schools of a selected educational division in Sri Lanka
Source: BMC Public Health. 2017 Jul 26;18:64. doi: 10.1186/s12889-017-4622-z (PMC5530461; doi:10.1186/s12889-017-4622-z)
Supplement: Supplementary file 2 — Assignment of score for the component on knowledge. (DOCX 14 kb) [file 12889_2017_4622_MOESM2_ESM.docx]

Additional File 2

As shown in Table S1 the 39 questions were in relation to ten components (subject area) on NCDs. Each component consisted of several items, which tested the knowledge on that subject area. Each item consisted of several statements the student had to answer “yes, no, or don’t know”. Each correct answer to the statement was given 1 and incorrect answer was given 0. Hence a student was able to score for each statement within the item in the component allowing a score of more than 1. Assignment of scores for the component on knowledge is as below.

**Table S1 – Assignment of score for the component on knowledge**

| **Subject area** | **Number of questions included** | **Total Score Assigned** |
| --- | --- | --- |
| **On Non Commumnicable Diseases (NCDs)** | | |
| Knowledge on NCDs in general | 2 | 11 |
| Diabetes mellitus | 5 | 31 |
| Hypertension | 4 | 24 |
| Ischemic heart disease | 5 | 23 |
| Stroke | 5 | 23 |
| Malignancies | 5 | 34 |
| **On risk factors** | | |
| Diet | 7 | 37 |
| Physical activity | 2 | 11 |
| Smoking | 2 | 10 |
| Alcohol | 2 | 12 |
| **Total score** | **39** | **216** |
